# Reinforcement of repressive marks in the chicken primordial germ cell epigenetic signature: divergence from basal state resetting in mammals

**DOI:** 10.1186/s13072-024-00537-7

**Published:** 2024-04-26

**Authors:** Clémence Kress, Luc Jouneau, Bertrand Pain

**Affiliations:** 1 Univ Lyon, Université Lyon 1, INSERM, INRAE, U1208, USC1361, Stem Cell and Brain Research Institute, Bron, France; 2 Université Paris-Saclay, UVSQ, INRAE, BREED, Jouy-en-Josas, 78350 France; 3 Ecole Nationale Vétérinaire d’Alfort, BREED, Maisons-Alfort, 94700 France

**Keywords:** Primordial germ cell, Epigenetic reprogramming, H3K9me3, 5mC, 5hmC, macroH2A, Chicken

## Abstract

**Background:**

In mammals, primordial germ cells (PGCs), the embryonic precursors of the germline, arise from embryonic or extra-embryonic cells upon induction by the surrounding tissues during gastrulation, according to mechanisms which are elucidated in mice but remain controversial in primates. They undergo genome-wide epigenetic reprogramming, consisting of extensive DNA demethylation and histone post-translational modification (PTM) changes, toward a basal, euchromatinized state. In contrast, chicken PGCs are specified by preformation before gastrulation based on maternally-inherited factors. They can be isolated from the bloodstream during their migration to the genital ridges. Our prior research highlighted differences in the global epigenetic profile of cultured chicken PGCs compared with chicken somatic cells and mammalian PGCs. This study investigates the acquisition and evolution of this profile during development.

**Results:**

Quantitative analysis of global DNA methylation and histone PTMs, including their distribution, during key stages of chicken early development revealed divergent PGC epigenetic changes compared with mammals. Unlike mammalian PGCs, chicken PGCs do not undergo genome-wide DNA demethylation or exhibit a decrease in histone H3 lysine 9 dimethylation. However, chicken PGCs show 5‑hydroxymethylcytosine loss, macroH2A redistribution, and chromatin decompaction, mirroring mammalian processes. Chicken PGCs initiate their epigenetic signature during migration, progressively accumulating high global levels of H3K9me3, with preferential enrichment in inactive genome regions. Despite apparent global chromatin decompaction, abundant heterochromatin marks, including repressive histone PTMs, HP1 variants, and DNA methylation, persists in chicken PGCs, contrasting with mammalian PGCs.

**Conclusions:**

Chicken PGCs’ epigenetic signature does not align with the basal chromatin state observed in mammals, suggesting a departure from extensive epigenetic reprogramming. Despite disparities in early PGC development, the persistence of several epigenetic features shared with mammals implies their involvement in chromatin-regulated germ cell properties, with the distinctive elevation of chicken-specific H3K9me3 potentially participating in these processes.

**Supplementary Information:**

The online version contains supplementary material available at 10.1186/s13072-024-00537-7.

## Background

Primordial germ cells (PGCs) are the embryonic precursors to the germ line. In mammals, their development involves extensive epigenetic changes affecting DNA methylation at the fifth position of cytosines (5mC) in CpG and post-translational modifications (PTMs) of histone proteins (reviewed in [1, 2]). This process represents an epigenome reprogramming, erasing inherited epigenetic information from gametes and preimplantation development before establishing new marks, such as parental imprints, for the next generation. In mice, PGCs are specified around embryonic day 6.25 (E6.25) from a subset of posterior proximal epiblast cells influenced by extra-embryonic ectoderm and endoderm signaling. Migration commences around E8, culminating in the arrival of PGCs at the genital ridges around E10.5. From E12.5 onward, sex-specific development occurs in the gonads, transforming PGCs into germline stem cells. The initial stages of epigenetic reprogramming occur during migration, characterized by a distinctive chromatin signature due to the genome-wide loss of dimethylation of histone H3 lysine 9 (H3K9me2) and an increase in trimethylation of histone H3 lysine 27 (H3K27me3) [3, 4]. Additionally, a global erasure of DNA methylation, including marks at imprinted loci, is initiated early [5] and continues until the 5mC level reaches its nadir in the gonads at about E13.5 [6, 7]. Changes in nuclear organization can be observed, particularly at chromocenters, the DAPI-dense foci where pericentric constitutive heterochromatin (PCH) forms clusters [4, 8, 9]. Indeed, higher-order genome organization, including chromosomal contact profiles and topologically associating domains, undergoes a maturation toward a globally euchromatinized state [10].

By E13.5, mouse PGCs attain a basal epigenetic state, probably crucial for mark resetting and conferring robust developmental capacity to the cells [11]. Global neutral chromatin states are also evident during the early stages of mammalian development, contributing to the plasticity of pluripotent stem cells (PSCs) with the capacity to initiate all lineages of the mature organism. Notably, embryonic stem cells (ESCs), derived from the mouse preimplantation embryo’s inner cell mass, exhibit a genome nearly devoid of repressive epigenetic marks and display a decondensed chromatin organization [12]. PGC specification and development involve a program activating or reactivating the expression of key pluripotency-associated genes, such as *OCT4*/*POU5F1*, *SOX2*, and *NANOG* [13]. Furthermore, PGCs can be converted in vitro to embryonic germ cells which share developmental properties with ESCs ([14] and the references therein). Conversely, PGCs can be reconstituted in vitro from PSCs [15, 16]. In mice, the resulting PGC-like cells erase genomic methylation during expansion and acquire a histone PTM profile similar to gonadal PGCs, allowing their use as a model for analyzing the involved epigenetic mechanisms [17].

Germ cell epigenome reprogramming appears conserved in mammals, as evidenced by postspecification epigenetic changes akin to those in mouse PGCs occurring in human [18–21], pig [22], and rabbit [23] PGCs, despite variations in specification mechanisms and gene expression programs. Nonmammalian vertebrates, such as *Xenopus*, zebrafish, and chickens, have been studied regarding PGC epigenetic marks, with a focus on DNA methylation (reviewed in [24]). Although results suggest genome-wide DNA demethylation does not occur, the presence of other major epigenetic changes remains plausible. Therefore, it is currently unclear whether germ cell epigenome reprogramming is genuinely absent in nonmammalian vertebrates, particularly in birds. Avian PGCs’ specification and gonadal migration differ from those of mammals, with the former using the preformation mode, where germ cells segregate early in embryogenesis due to localized maternally-inherited determinants before or immediately following fertilization [25]. In chickens, PGCs are initially observed in the central area of the blastoderm in the unincubated embryo (stage EG&K X) [26]. During gastrulation, they move to the anterior part of the embryo termed the germinal crescent (stage HH4; refer to “Methods” section for staging). Following vascular system formation, they migrate to the blood circulation and eventually reach the genital ridges, where most are settled at HH16 (about 55 h). Proliferation and sex-specific differentiation lead to PGCs and surrounding somatic cells forming the gonads (for a review of chicken PGC development, refer to [27]). PGCs and their developmental processes are important in avian research, primarily owing to their suitability for in vitro genetic modification and the generation of transgenic animals. Although pluripotent cells similar to mammalian ESCs have been derived from the blastoderm in chickens, their efficacy in producing germline chimeras is limited [28]. PGCs can be readily isolated from the blood and thereafter cultured, genetically modified, and cryopreserved. Upon reintroduction into a recipient embryo at the appropriate development stage, they effectively colonize the gonads, transforming into functional germline stem cells [29]. Given these properties, PGCs also play a crucial role in the conservation of avian genetic resources.

Our previous research explored global histone PTMs and DNA methylation in chicken cultured migratory PGCs (cPGCs) compared with somatic undifferentiated and differentiated cell types, revealing cPGCs’ distinctive epigenetic profile [30]. Notably, the mammalian PGC epigenetic signature, featuring low 5mC, low H3K9me2, and high H3K27me3, was absent, whereas H3K9me3 levels surpassed those in somatic cell types. Additionally, chicken cPGCs were clearly epigenetically distinct from chicken PSCs, exemplified by blastodermal cells and in vitro-derived ESCs, similar in transcriptome and epigenetic profile [31]). This observation suggested that the mammalian model of epigenomic resetting to a basal, PSC‑like state does not apply to chickens. However, the existence of this signature in vivo remained unconfirmed, leaving the question of whether chicken PGCs undergo extensive epigenome reprogramming, similar to mammals, unanswered. Thus, in the present study, we investigated the global epigenetic profile of chicken germ cells during early embryo development, aiming to better understand its characteristics and potential role by comparing it with known mammalian data.

## Methods

### Sample collection

Chicken cPGCs, ESCs, and embryonic fibroblasts were obtained as described previously [30]. Embryos were collected from fertilized eggs (JA57 strain), and their developmental stages were assessed according to EG&K [32] and HH [33] sequences. Up to stage HH23 (incubation of approximately 4 days), whole embryos were fixed. For 6‑ to 14‑day‑old embryos, left gonads were dissected together with mesonephros and fixed. All samples underwent phosphate-buffered saline (PBS) washing and fixation in 4% paraformaldehyde in PBS at room temperature (RT), followed by three further PBS washes. Fixation durations were as follows: 10 min for cultured cells, 15 min for stage ≤ HH4 embryos, and 30 min for stage ≥ HH13 embryos and gonads. Cryosections of gonads and stage ≥ HH16 embryos were prepared as described previously [30]. Sexing was achieved by PCR [34] using genomic DNA extracted from embryonic tissue with the Wizard DNA Purification Kit (Promega).

### Fluorescence immunodetection

All steps were performed at RT unless otherwise specified. Samples were permeabilized for 30 min with Sca*l*eCUBIC‑1 clearing solution [35] for tissue sections and with 0.5% Triton X‑100 in PBS for whole embryos and cultured cells, followed by three PBS washes. Saturation was conducted for 1 h using 2% bovine serum albumin in PBS (blocking solution). Samples were incubated with primary antibodies overnight at 4 °C, washed with 0.1% Tween‑20 in PBS three time for 10 min per wash, and incubated with fluorescence-coupled secondary antibodies for 1 h at RT. Antibodies were diluted in the blocking solution (references and dilutions are given below). For 5mC and 5hmC detection, acid-induced epitope unmasking was performed, following permeabilization, by incubation in 4 N HCl for 1 h at 37 °C, after which washes in 100 mM Tris‑HCl (pH 8) and PBS were performed. Germ cells were identified using labelling with rabbit antibodies against DAZL (for whole embryos, stage ≥ HH16 embryo sections, and gonad sections) or CVH (for gonad sections only). For one experiment (H3K9me3 combined to HP1 and DNA detection), as it was technically not possible to add the germ-specific antibody, germ cells were identified using their specific H3K9me3 enrichment and nuclear morphology, based on previous analyses including germ cell markers. Primary antibodies were incubated together when they were from different species. When the antibody against the epigenetic mark was produced in rabbits, germ cell marker detection preceded as follows: initial incubation with the antibody against the mark, followed by the fluorescence-coupled secondary antibody (Fab fragment), then blocking of any remaining free rabbit IgG sites through incubation with 30 µg/mL of anti-rabbit Fab fragment (Jackson ImmunoResearch, 711-007-003), and finally germ-specific labelling. Regarding 5mC and 5hmC staining of whole embryos, germ cell labelling using the anti-DAZL antibody was performed before denaturation, followed by 5 min of postfixation in paraformaldehyde to achieve optimal germ cell detection. DNA was counterstained for 30 min using 1 μM TO‑PRO‑3 (Molecular Probes) in PBS, and samples were mounted with SlowFade Diamond antifade reagent (Invitrogen). As no satisfactory DNA dye was found for the denatured tissue samples, nuclear contours were located using other fluorescent signals (epigenetic mark, RNA Pol II, or germ cell labelling).

The following primary antibodies were used at the indicated dilutions: anti-H3K9me3 (1/500, ab8898), anti-H3K4me3 (1/500, ab8580), anti-H3K4me1 (1/500, ab8895), anti-H3K27ac (1/500, ab4729), anti-macroH2A1 (1/500, ab183041), anti-DAZL (1/500, ab34139), anti-DAZL (1/500, ab215718), all rabbit (Abcam); anti-macroH2A1 (1/500, ab91528), mouse (Abcam); anti-H3K9me3 (1/1000, 39161), anti-H3K9me2 (1/500, 39753), anti-H3K9ac (1/500, 39917), anti-5hmC, (1/1000, 39769), all rabbit (Active Motif); anti-5mC (1/500, BI-MECY-0100), mouse (Eurogentec); anti-RNA Pol II (1/200, 1PB-7G5), mouse (Euromedex); anti-H3K27me3 (1/500, 07-449), rabbit (Millipore); anti-HP1beta (1/250, 1MOD-1A9) and anti-HP1gamma (1/250, 2MOD-1G6), mouse (Millipore); anti-CVH (1/1000, VN1), custom-made rabbit (Biotem); and anti-CENP‑T (1/1000), rabbit (gift from T. Fukagawa). All primary antibodies raised against mammalian chromatin proteins have been validated for the chicken corresponding proteins using Western blot on chicken cell extracts. The following secondary antibodies from Jackson ImmunoResearch were used at 1/500 dilutions: anti-rabbit IgG (111-547-003) and anti-mouse IgG (115-546-146) conjugated with Alexa Fluor 488 (1/500), anti-rabbit IgG (711-167-003) conjugated with Cy3, and anti-mouse IgG (715-605-150) conjugated with Alexa Fluor 647.

### Transmission electron microscopy

Gonads dissected from 14‑day-old embryos were fixed in 2% glutaraldehyde (Electron Microscopies Sciences) overnight at 4 °C, followed by rinsing three times at 4 °C. Subsequently, tissues were dehydrated in a graded ethanol series and transferred to propylene oxide (EMS). Impregnation was performed with Epon epoxy resin (EMS). Inclusion was achieved by polymerization at 60 °C for 72 h. Ultrathin sections (approximately 100 nm thick) were cut on a UC7 (Leica) ultramicrotome, mounted on 200 mesh copper grids, and contrasted with uranyl acetate and lead citrate (EMS). Sections were examined using a Jeol 1400JEM (Tokyo, Japan) 120 kV transmission electron microscope equipped with a Gatan Orius 1000 camera in the wide-field position, along with Digital Micrograph software (Gatan Inc.).

### Image analysis and quantification

Fluorescently labeled nuclei in single-plane images were captured using a Leica DM 6000 CS SP5 or TCS SPE confocal laser-scanning microscope equipped with a × 63/1.4 NA oil immersion objective in sequential scanning mode, with settings applied to avoid signal saturation. Fiji [36] was used for image processing. Epigenetic mark staining in tissue sections were quantified as follows. Nuclei in each image containing germ and somatic cells were initially segmented based on the DNA counterstain signal using the MorphoLibJ plugin [37], with manual correction applied if necessary. Nuclei visible in their equatorial plane were selected for quantification. Total fluorescence intensities for the epigenetic mark and DNA were measured for each nucleus section, and the epigenetic mark per amount of chromatin was calculated as the ratio of these intensities. An average of 41 germ cells (min 13) and 146 somatic cells (min 30) at each time point were used for quantification. Each nucleus’ ratio was then divided by the mean value of the ratios for somatic cells in the image, yielding the normalized intensity that allowed pooling of results from different images. For DNA methylation analysis, where DNA staining was unavailable, nuclei were segmented using the epigenetic mark or the germ cell marker antibody signal. Chromatin per unit of nuclear area was obtained from chromatin condensation quantification of matching stage and sex samples. For chromatin condensation quantification, DNA counterstain fluorescence intensity per unit area was measured for each nucleus and normalized, as described above, using the mean value for somatic cells in the image.

The radial distribution of epigenetic marks and DNA labelling in nuclei was quantified using eroded volume fraction analysis applied to nuclei sections. The 3D suite plugin [38] from Fiji was employed to divide nuclei into 25 concentric sections of equal area and measure the mean signal intensity for each section. Subsequently, section intensities were expressed as percentages of the total intensity in the nucleus.

To evaluate the enrichment for H3K9me3 or CENP‑T at macroH2A1 foci, these foci were segmented using morphological and top hat filters from the MorphoLibJ plugin of Fiji. The mean labelling intensity of the codetected protein was measured in each segmented area and expressed relative to the mean intensity in the whole nucleus. In total, about 15 nuclei, giving > 100 spots, were used for each analysis.

### RNA extraction and RNA-seq

Total RNA from cultured cells was extracted using TRIzol reagent (Life Technologies), according to the manufacturer’s instructions. Three and two biological replicates were analyzed for cPGC and ESCs, respectively. RNA libraries were generated using 1 µg of total RNA and sequenced using Illumina reagents, protocols, and instruments (NextSeq500 or HiSeq2500) by Helixio (https://www.helixio.fr/) or Eurofins Genomics (https://www.eurofinsgenomics.eu/) to obtain paired-end reads (75 or 100 bp).

### ChIP and ChIP-seq

Cells were fixed for 5 min at 37 °C with 1% formaldehyde by adding 10× crosslinking solution [11% formaldehyde, 0.1 M NaCl, and 50 mM Hepes (pH 8)] to the cell culture medium. The crosslinking reaction was stopped with a 2 min incubation after adding glycin at 0.125 M in the medium. After a PBS wash, the cells were kept at 4 °C and collected using scrapping or centrifugation in chromatin wash solution 1 [10 mM Hepes (pH 8), 10 mM EDTA, 0.5 mM EGTA, 0.25% Triton X‑100, and protease inhibitor cocktail]. Following a 10 min wash, the cells were centrifugated, resuspended and washed in chromatin wash solution 2 [10 mM Hepes (pH 8), 2 mM EDTA, 0.5 mM EGTA, 200 mM NaCl, and 0.01% Triton] and then in chromatin wash solution 3 [25 mM Tris–HCl (pH 8), 2 mM EDTA, 150 mM NaCl, and 0.1% SDS] for 10 min at 4 °C in each solution. The lysed cells were resuspended at 10^7^ cells/mL in sonication buffer [25 mM Tris–HCl (pH 8), 2 mM EDTA, 150 mM NaCl, and 0.5% SDS] and sonicated in an ice bath using a Bioruptor (Diagenode) at full power for 15 cycles (30 s on + 30 s off). Sheared chromatin was frozen at − 80 °C after adjusting the Triton concentration to 1%. Crosslink reversion (200 mM NaCl at 65 °C overnight) followed by DNA extraction (RNase incubation, phenol–chloroform extraction, and ethanol precipitation) were performed using an aliquot of chromatin to confirm that DNA fragment size was ≤ 500 bp and to measure DNA concentration.

Immunoprecipitation was performed using an amount of chromatin corresponding to 10 µg of DNA. Chromatin was diluted in 250 µL of ChIP Buffer [25 mM Tris–HCl (pH 8), 2 mM EDTA, 150 mM NaCl, 0.5% SDS, and 1% Triton X‑100] and incubated overnight at 4 °C with 1 µg of H3K9me3 antibody (Abcam, ab8898). A chromatin sample was also incubated without antibody to estimate background binding. For antibody-bound chromatin collection, 10 µL of Protein A magnetic beads (Dynabeads, Life Technologies), preincubated with 2.5 µg of sheared salmon sperm DNA and 10 µg of bovine serum albumin in TE, were added to the sample and incubated for 2 h at 4 °C. Subsequently, the beads were washed for 5 min per wash with ChIP wash solution 1 [25 mM Tris–HCl (pH 8), 2 mM EDTA, 150 mM NaCl, 0.1% SDS, and 1% Triton X‑100], solution 2 [25 mM Tris–HCl (pH 8), 2 mM EDTA, 500 mM NaCl, 0.1% SDS, and 1% Triton X‑100], solution 3 [10 mM Tris–HCl (pH 8), 1 mM EDTA, 250 mM LiCl, 1% NP‑40, and 1% Na deoxycholate], and TE. Chromatin was released from the beads through incubation in 300 µL of denaturation solution [10 mM Tris–HCl (pH 8), 1% SDS, and 200 µg/mL proteinase K], for 2 h at 37 °C. DNA was then purified using phenol–chloroform extraction and ethanol precipitation and resuspended in 100 µL of TE. A 2 µL sample was used to assess the efficiency of immunoprecipitation through quantitative PCR using the StepOnePlus Real-Time PCR System and Fast SYBR® Green Master Mix (Applied BioSystems). For this purpose, sequences from housekeeping genes, inactive genes, heterochromatic loci, and repeated elements were quantified in immunoprecipitated (IP), mock-IP, and input chromatin samples. Libraries were prepared for IP and input DNA (5 ng) using the NEBNext® Ultra™ Library Prep Kit, followed by sequencing with the NextSeq 500 High Output v2 Kit on a NextSeq500 sequencing system (Illumina) by Helixio (https://www.helixio.fr/) to obtain paired-end reads (75 bp).

### RNA-seq analysis

Reads were aligned to the chicken transcriptome [Ensembl gene transcripts for *Gallus gallus* 5 (galGal5)] using the splice junction mapper TopHat [39] in conjunction with the short-read aligner Bowtie 2 [40]. The DESeq2 R package [41] was employed for differential expression analysis on count tables obtained using FeatureCounts [42]. To assess transcript relative abundance, TPMs were computed for each biological sample using Salmon [43]. The TPM value for each gene was calculated as the sum of the TPM values of the corresponding transcripts. Final gene TPMs for each cell type are represented by the mean values of the biological replicates. To visualize transcriptional activity in the UCSC genome browser, local coverage was obtained from the read alignment files using genomecov from Bedtools [44] and displayed as a density track (mean of the replicate values).

### ChIP-seq analysis

Sequence analysis was performed on the chicken genome version 5 (galGal5). Coordinates and annotations of genes (Ensembl genes) and repeated elements (RepeatMasker output) were downloaded from the UCSC table browser. Following quality control, sequencing reads were aligned using Bowtie 2 with its default parameters.

The percentage of bases in immunoprecipitated DNA located in repeated elements was assessed as the ratio between the coverage of the repeated element compartment and the coverage of the whole genome in sequencing libraries. The repeated element compartment of the genome was defined by aggregating all elements from RepeatMasker with a size ≥ 50 bp. Coverages were calculated using Samtools depth [45]. The presence of CNM repeats in sequencing libraries was determined by the unsupervised identification of satellite repeats using TAREAN [46] with its default parameters. Detailed analysis of enrichment at repeated elements was performed using the RepEnrich method [47]. The pseudogenome was set up using the list of elements identified through RepeatMasker, excluding the “unspecified,” “unknown,” and “ARTEFACT” categories, and retaining only the telomeric repeats from the “simple repeat” category. Counts for each element type in the H3K9me3-immunoprecipitated and input DNA libraries were determined using RepEnrich2. For normalization, counts were divided by the total number of reads in the libraries. The IP/Input ratios were calculated for each element type prior to grouping based on repeat class.

Analysis of H3K9me3 local enrichment focused on the nonrepetitive part of the genome. Assessments of repeated element enrichments are not reliable owing to multimapping sequencing reads generated by repeated sequences, making unambiguous assignment to a genome position challenging. Consequently, for a more accurate understanding of local enrichment at unique sequences, we detected H3K9me3 peaks after filtering out ambiguous reads. Reads in proper pairs with a mapping quality ≥ 30, indicating mapping to a unique location, were selected using Samtools. Initial peak calling was performed based on the comparison of immunoprecipitated and input (control) alignment files using MACS2 [48], selecting the options broad regions and cutoff 0.1 for the callpeak command. Fold enrichment for H3K9me3 along the genome was calculated using the MACS2 bdgcmp command, and the output bedGraph file was converted to bigWig format for visualization in the UCSC genome browser. SICER [49] was used to delineate islands with its default parameters, with the exceptions of window and gap size, which were set to 300 and 1500, respectively. The final list of H3K9me3 domains included all the islands with a false-discovery rate < 10^–10^ and those at least partially overlapped by MACS2 peaks among islands with a higher false-discovery rate. For practical reasons, the analysis was executed using genomic sequences corresponding to the main scaffolds of autosomes (hereafter, referred to as the “whole genome”). To compare the distribution of H3K9me3 domains between cell types, we used Bedtools. Each base in a domain was categorized as “in shared core” if it was in a domain segment found in both cell types, “in expansion” if it was in a cell-specific segment prolongating a shared domain, and “in cell type-specific domain” if it was in a domain without any overlap in the other cell type.

The functional regions of the genome were defined using gene coordinates from Ensembl, with the repeated elements defined through RepeatMasker. Definitions were as follows: whole genome: defined above; promoters: 1 kb centered on TSS; genes: gene bodies (TSS to TTS); intergenic regions: whole genome, excluding the promoters and gene bodies; repeated elements: RepeatMasker elements with a size ≥ 50 bp. The coverage values, especially those of repeated elements, may be underestimated because multimapping reads are not considered. The number of bases covered by H3K9me3 domains for each type of region was calculated using Bedtools and divided by the total number of bases of the region. A similar coverage calculation was performed for single gene analysis (including the promoter). Functional annotation analysis of genes covered by H3K9me3 domains was performed using DAVID [50].

Data handling, plotting, and statistical analysis were conducted using R (R Core Team, 2022; https://www.R-project.org/) and DataGraph (Visual Data Tools, Inc. Chapel Hill, NC, USA; https://www.visualdatatools.com/).

## Results

### Transcriptome analysis of cPGCs

To correlate in vivo observations of epigenetic marks with epigenetic modifier expression in early chicken embryo germ cells, we elucidated germ cell-specific expression profiles. Comparisons were made between cPGCs and ESCs, the in vitro derivatives of early embryonic germ and somatic cells, respectively. RNA-seq, followed by differential gene expression analysis and transcript per million (TPM) analysis, was performed for a comprehensive estimation of gene expression levels (Additional file 1: Table S1; excerpt in Additional file 2: Table S2). As expected, pluripotency-associated genes, such as *NANOG*, *OCT4/POUV*, *SOX2*, *SOX3*, *KLF2*, *KLF4*, and *KLF5*, exhibited robust expression in both cell types. Differential analysis confirmed significantly higher expression of germinal genes, including *DAZL*, *DDX4*, *PIWIL1*, and *MAEL*, in cPGCs. Interestingly, *HOX* genes were markedly repressed in cPGCs, with a mean TPM of 0.05 compared with 6.61 in ESCs. This repression aligns with observations in mammals, where *HOX* genes are downregulated in migrating PGCs compared with somatic neighboring cells, likely reflecting the necessity to inhibit somatic transcriptional programs for germ specification [13, 14].

### DNA methylation in chicken gonadal PGCs

**Fig. 1:**
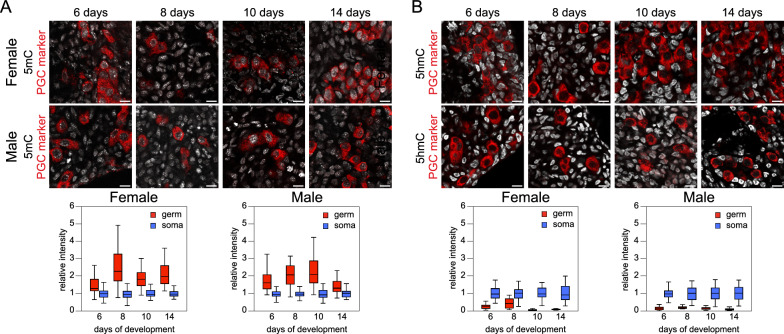
5mC and 5hmC in chicken embryo gonads. **A** Immunodetection of 5mC (gray) and germ cell marker (red) in tissue sections. Scale bar: 10 µm. Quantification of fluorescence intensity in germ and somatic cell nuclei is shown below. Number of analyzed nuclei in 6‑, 8‑, 10‑, and 14‑day-old embryos: for females, 44, 50, 60, and 54 germ cells and 74, 101, 68, and 57 somatic cells, respectively; for males, 28, 22, 56, and 34 germ cells and 71, 76, 76, and 47 somatic cells, respectively. **B** Immunodetection of 5hmC (gray) and germ cell marker (red) in tissue sections. Scale bar: 10 µm. Quantification of fluorescence intensity in germ and somatic cell nuclei is shown below. Number of analyzed nuclei in 6‑, 8‑, 10‑, and 14‑day-old embryos: for females, 48, 27, 49, and 34 germ cells and 76, 81, 54, and 41 somatic cells, respectively; for males, 37, 13, 39, and 33 germ cells and 61, 46, 42, and 48 somatic cells, respectively

In mammals, DNA methylation in PGCs reaches its nadir upon settling at the genital ridges and proliferating in the developing gonads. Therefore, we examined postmigratory chicken PGCs in the embryonic gonads during the period of germ and somatic gonadal cell proliferation, before female germ cells initiate meiotic arrest after approximately 14 days of development [51]. Fluorescence immunodetection in tissue sections of the gonads from 6‑, 8‑, 10‑, and 14‑day-old chick embryos of both sexes was used to visualize epigenetic marks. Germ cells were identified by the presence of CVH/DDX4 and DAZL proteins as germ-specific markers in early chicken embryos [52, 53]. Fluorescent signals were observed in the nuclei of these germ cells and surrounding somatic cells. At all stages and for both sexes, 5mC was clearly detected in germ cells and neighboring somatic cells (Fig. 1A). Signal quantification revealed consistently higher global 5mC levels in germ cells compared with somatic cells, contrasting with the global DNA demethylation observed in mammalian gonadal PGCs. Regarding DNA hydroxymethylation on CpG, a pronounced reduction in 5hmC has previously been observed in mouse and human gonadal germ cells, with this decrease following a transient increase upon the arrival of mouse PGCs at the genital ridges [9, 20]. In chicken gonads, 5hmC was abundant in somatic cells but barely detectable in germ cells, regardless of stage and sex (Fig. 1B). Importantly, the global 5mC and 5hmC levels in gonadal PGCs were similar to those quantified previously in cPGCs relative to somatic cells [30].

Expression of DNA methylation modifiers in germ cells (Additional file 2: Table S2) was explored by comparing cPGCs to ESCs, which exhibit 5mC and 5hmC levels typical of somatic cell types [30]. In mouse PGCs, reduced expression or protein presence is observed for several factors involved in 5mC maintenance, including DNA methyltransferases (DNMTs) and UHRF1/NP95, whereas TET1 and TET2 enzymes, converting to 5mC to 5hmC, showed increases [3, 5, 7, 13, 54]. In chicken cPGCs, we found that DNMT genes were not underexpressed, aligning with previous reports indicating higher expression of *DNMT1*, *DNMT3A*, and *DNMT3B* in PGCs compared with somatic gonadal cells [55]. *UHRF1* exhibited slightly higher expression in cPGCs relative to ESCs, whereas *TET1*, *TET2*, and *TET3* showed lower expression, suggesting a balance that does not favor DNA demethylation. Notably, *LSH*/*HELLS*, necessary for repetitive element methylation, especially at PCH [56], showed higher expression in cPGCs, consistent with the presence of large nuclear foci of 5mC (Fig. 1A).

### Histone PTMs in chicken gonadal PGCs

**Fig. 2:**
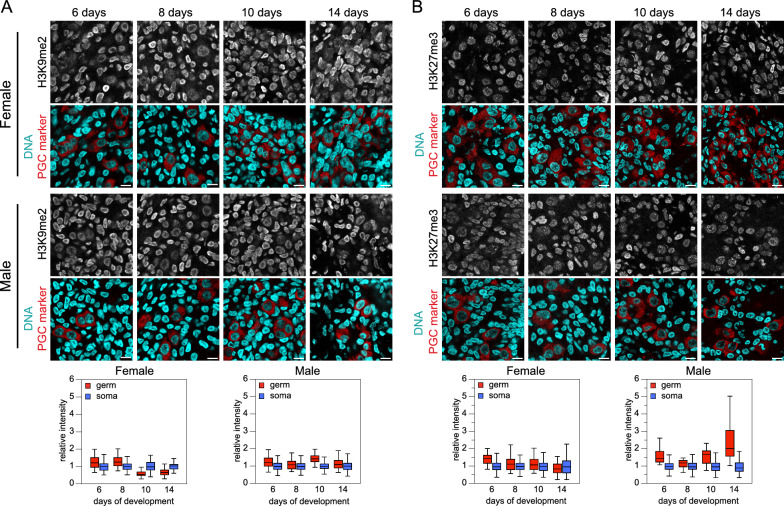
H3K9me2 and H3K27me3 in chicken embryo gonads. **A** Immunodetection of H3K9me2 (gray) and germ cell marker (red) in tissue sections. DNA staining (cyan). Scale bar: 10 µm. Quantification of fluorescence intensity in germ and somatic cell nuclei is shown below. Number of analyzed nuclei in 6‑, 8‑, 10‑, and 14‑day-old embryos: for females, 24, 29, 52, and 45 germ cells and 133, 179, 65, and 56 somatic cells, respectively; for males 33, 34, 41, and 38 germ cells and 125, 126, 104, and 112 somatic cells, respectively. **B** Immunodetection of H3K27me3 (gray) and germ cell marker in tissue sections. DNA staining (cyan). Scale bar: 10 µm. Quantification of fluorescence intensity in germ and somatic cell nuclei is shown below. Number of analyzed nuclei in 6‑, 8‑, 10‑, and 14‑day‑old embryos: for females, 42, 48, 34, and 84 germ cells and 411, 329, 202, and 314 somatic cells, respectively; for males, 31, 21, 34, and 44 germ cells and 434, 224, 225, and 244 somatic cells, respectively

To determine whether certain histone-related events of mammalian epigenome reprogramming occurred in chicken germ cells, we analyzed H3K9me2 and H3K27me3, the heterochromatic histone PTMs that exhibit the most pronounced changes in mammalian PGCs. In females, the H3K9me2 level was slightly higher in germ cells than in somatic gonadal cells initially, then gradually decreased (by approximately twofold), whereas in males, it remained consistently higher at all stages (Fig. 2A). Thus, the enduring loss of H3K9me2 observed in mammalian gonads [3, 4, 20, 21] did not manifest in chickens. Initially, the H3K27me3 level was marginally higher in germ cells than in somatic gonadal cells in females but was consistently higher (up to 2.4-fold) in males (Fig. 2B). Chromatin enrichment for H3K27me3 occurs transiently in human PGCs [20, 21] and transiently or for an extended period, depending on the study, in mouse PGCs [3, 4]. Our results suggest the presence of sex-specific variations in H3K27me3 levels in chicken PGCs. However, the observed changes in H3K9me2 and H3K27me3 were not consistent with the hypothesis of the latter replacing the former, as proposed in the mammalian model [3].

**Fig. 3:**
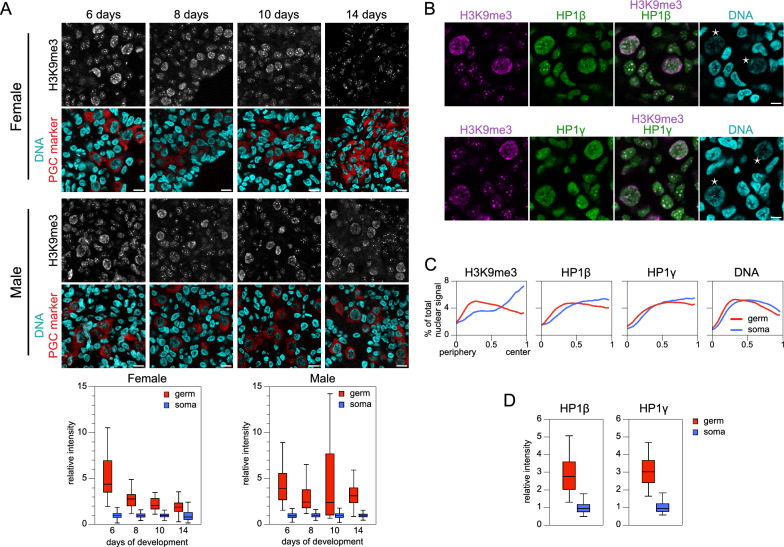
Constitutive heterochromatin marks and their nuclear distribution in gonadal germ cells. **A** H3K9me3 in chicken embryo gonads. Immunodetection of H3K9me3 (gray) and germ cell marker (red) in tissue sections. DNA staining (cyan). Scale bar: 10 µm. Quantification of fluorescence intensity in germ and somatic cell nuclei is shown below. Number of analyzed nuclei in 6‑, 8‑, 10‑, and 14‑day-old embryos: for females, 61, 53, 34, and 91 germ cells and 685, 423, 311, and 210 somatic cells, respectively; for males, 88, 46, 64, and 40 germ cells and 377, 266, 183, and 229 somatic cells, respectively. **B** Immunodetection of H3K9me3 (magenta) and HP1beta and gamma (green) in a 10‑day-old male embryo gonadal tissue section. Stars indicate germ cell nuclei, identified using their specific H3K9me3 enrichment and nuclear morphology. DNA staining (cyan). Scale bar: 5 µm. **C** Analysis of the radial distributions of H3K9me3, HP1, and DNA signal intensities in germ and somatic cell nuclei. Number of analyzed nuclei for germ and somatic cells: 44 and 55 for H3K9me3, 44 and 55 for DNA, 25 and 30 for HP1alpha, and 19 and 25 for HP1gamma, respectively. **D** Quantification of fluorescence intensity for HP1beta and gamma immunodetection in gonadal germ and somatic cell nuclei from 10-day-old male embryos. Number of analyzed nuclei for germ and somatic cells: 27 and 60 for HP1beta, and 19 and 45 for HP1gamma, respectively

Next, we examined H3K9me3, the histone PTM specific for constitutive heterochromatin. Remarkably, the global level of H3K9me3 was substantially higher in chicken gonadal germ cells compared with surrounding somatic cells (Fig. 3A). This enrichment surpassed that observed for H3K9me2 and H3K27me3, being markedly higher (2.0 to 4.5-fold) and observed in both sexes at all stages. This result aligns with our previous observation that chicken cPGCs exhibit a high level of H3K9me3 compared with several somatic cell types [30]. However, it contrasts with findings in mammals, where H3K9me3 levels are known to be low in germ cells or similar to those in surrounding gonadal cells [4, 20, 21, 57]. According to the present RNA-seq analysis and our previous findings [30], *KMT1B/SUV39H2*, encoding the main enzyme responsible for H3K9 trimethylation in heterochromatin, was more expressed in PGCs than in ESCs and several other somatic cell types, potentially contributing to the observed high H3K9me3 level. Moreover, several genes encoding enzymes involved in H3K9 trimethylation deposition or removal were more highly expressed in chicken PGCs than in ESCs, with *KDM4C* being significantly overexpressed, and *KMT1E*, *KDM3A*, *KMT1D*, *KDM4B* and *KDM7A* exhibiting slightly higher expression levels (Additional file 2: Table S2).

We also examined several histone PTMs specific to active chromatin: H3K4me3, H3K4me1, H3K27ac, and H3K9ac. Regarding H3K4 methylation, germ cell levels did not markedly differ from somatic gonadal cell levels, with a decreasing tendency observed in females. In males, germ cell H3K4 methylation levels remained higher than those in somatic cells at all stages (Additional file 2: Fig. S1A and B). Regarding acetylation, H3K27 in germ cells followed a similar trajectory in both sexes, starting at similar levels relative to somatic cells and ending at lower levels (Additional file 2: Fig. S1C). H3K9 acetylation constantly maintained significantly lower levels in germ cells compared with somatic cells (Additional file 2: Fig. S1D). These observations resembled those documented for gonadal mouse PGCs, characterized by low levels of active histone PTMs [4], as opposed to findings reported for human PGCs [21].

### Nuclear organization and chromatin density in chicken PGCs

As mammalian PGCs differ from somatic cells in the abundance and nuclear distribution of epigenetic marks and architectural proteins, particularly at chromocenters, we investigated whether this distinction extended to chicken PGCs. Initially, we examined the presence of epigenetic marks at PCH, which forms chromocenters in chicken cPGC nuclei [30], on gonad tissue sections. In chicken gonadal PGCs, 5mC displayed a typical pattern of enrichment at chromocenters, similar to somatic cells (Fig. 1A). Although 5hmC was barely visible in PGC nuclei, overexposure after immunodetection revealed a faint modification without the relatively homogenous pattern found in somatic cells. Rather, it was concentrated in large foci (not shown), akin to chromocenters, reminiscent of the transient enrichment observed in mouse gonadal PGCs [9]. Regarding repressive histone PTMs, H3K9me3 was strongly enriched at chromocenters in PGCs, whereas H3K9me2 was unenriched, not differing from the H3K9me2 levels observed in somatic cells (Fig. 2A). Some of the largest chromocenters in gonadal PGC nuclei were enriched in H3K27me3, as previously observed for cPGCs and chicken PSCs [30]. Surprisingly, they also exhibited enrichment of H3K4me3, indicative of a unique type of PCH, perhaps related to bivalent chromatin (not shown). Therefore, no loss of heterochromatin marks at PCH, as initially reported for mouse PGCs [4], was observed. On the contrary, heterochromatin marks were present at PCH in chicken gonadal PGCs, consistent with later reports for mouse and human PGCs [20, 57].

We examined the nuclear distribution of heterochromatin components in greater detail to understand which genome compartments were affected by the global enrichment of H3K9me3 in PGCs. In somatic cells, H3K9me3 was concentrated in large foci corresponding to centrally located bright chromocenters, typical of constitutive PCH. In germ cells, H3K9me3 was more homogeneously distributed in the nucleoplasm, suggesting its presence in other, more diffuse, regions as well as PCH (Fig. 3B). Heterochromatin proteins HP1beta and gamma were mainly enriched at chromocenters in both cell types (Fig. 3B). Some large peripheral H3K9me3-rich domains in germ cells were not enriched for HP1, suggesting a chromatin type wherein H3K9me3 plays a role other than constitutive heterochromatin maintenance. The radial distributions of these heterochromatin components in nuclei confirmed differences between the two cell types (Fig. 3C). Maximal H3K9me3 enrichment was at the nuclear center in somatic cells but near the periphery in PGCs. HP1 isoforms’ radial distributions were similar between the two cell types, with a slight shift to the periphery observed for PGCs. Notably, heterochromatin proteins HP1beta and gamma were more abundant in chicken germ cell nuclei than in somatic cell nuclei (Fig. 3D), thereby differing from mammalian PGCs, in which HP1 isoforms in the nuclei tend to be reduced or absent during PGC epigenetic reprogramming [4, 20, 57]. Overall, the high levels and distributions of heterochromatin components in chicken PGCs did not correspond to a mere intensification of the somatic cell pattern, suggesting the germ-specific functions of these epigenetic actors.

**Fig. 4:**
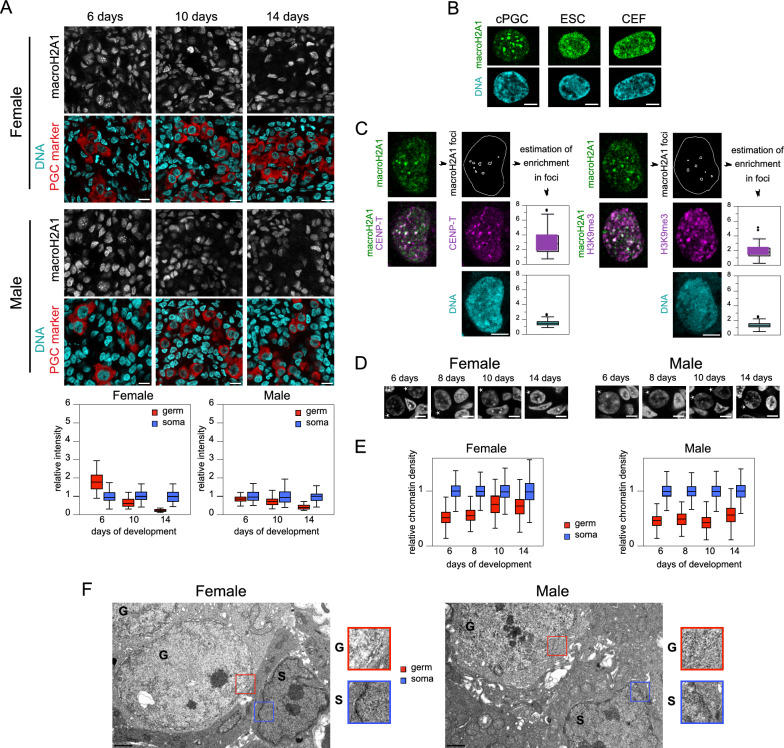
MacroH2A1 and chromatin density in chicken embryo germ cells. **A** Immunodetection of macroH2A1 (gray) and germ cell marker (red) in gonad tissue sections. DNA staining (cyan). Scale bar: 10 µm. Quantification of fluorescence intensity in germ and somatic cell nuclei is shown below. Number of analyzed nuclei in 6‑, 10‑, and 14‑day-old-embryos: for females, 43, 44, and 38 germ cells and 76, 45, and 30 somatic cells, respectively; for males, 25, 39, and 34 germ cells and 46, 60, and 60 somatic cells, respectively. **B** macroH2A1 nuclear distribution in chicken germ and somatic cells. Immunodetection of macroH2A1 (green) in cPGCs, ESCs, and CEFs. DNA staining (cyan). Scale bar: 5 µm. **C** macroH2A1 foci compared to pericentric heterochromatin. Immunodetection of macroH2A1 (green) and CENP‑T or H3K9me3 (magenta) in the nucleus of cPGCs. DNA staining (cyan). Scale bar: 5 µm. Box plots show the enrichment in CENP‑T and H3K9me3 (mean intensity in each focus compared to mean intensity in whole nucleus) for more than 100 foci. **D** Representative germ and somatic cell nuclei in gonadal tissue sections with DNA staining (gray). Stars indicate germ cell nuclei. Scale bar: 5 µm. **E** Quantification of chromatin density (measured as the intensity of the DNA staining by unit area) in germ and somatic cell nuclei. Number of analyzed nuclei in 6‑, 8‑, 10‑, and 14‑day-old embryos: for females, 311, 284, 308, and 489 germ cells and 1873, 1754, 1101, and 919 somatic cells, respectively; for males, 323, 241, 323, and 316 germ cells and 1899, 1220, 924, and 1069 somatic cells, respectively. **F** Ultrastructure of germ and somatic cell nuclei observed using transmission electron microscopy in 14‑day-old embryo gonads. Nuclei of germ (G) and somatic (S) cells are indicated. Scale bar: 1 µm. Magnified views of the nuclear envelope and the associated chromatin are shown

We also investigated the presence of macroH2A, a histone variant that undergoes marked redistribution during mammalian PGC reprogramming, including concentration at PCH or depletion from the nucleus [4, 20]. Notably, macroH2A1, a variant stabilizing nucleosome in chicken cells [58], predominantly localized to large nuclear foci in gonadal germ cells at early stages compared with somatic cells, becoming barely detectable later (Fig. 4A). This distinctive nuclear distribution was also observed in cPGCs but not in cultured somatic cells, such as ESCs and chicken embryonic fibroblasts (CEFs) (Fig. 4B). Consistently, chicken cPGCs exhibited low levels of macroH2A1 mRNA (Additional file 2: Table S2) and protein (not shown) compared with ESCs. To confirm that macroH2A1 foci in chicken PGCs corresponded to PCH, we examined the presence of other PCH characteristics in cPGC nuclei (Fig. 4C). At macroH2A1 foci, the centromere protein CENP‑T was, on average, threefold more concentrated than in the whole nucleus, indicating proximity to centromeres. H3K9me3 and DNA were 1.9-fold and 1.4-fold more concentrated, respectively, suggesting that macroH2A1 foci tended to contain heterochromatin.

Subsequently, we examined indicators of chromatin density in chicken PGCs, searching for possible “loosening,” similar to the epigenome reprogramming of mouse PGCs. Chromocenters have been reported to disappear around E10.5 [4] or become less visible [8, 10, 57] in mouse PGCs. Although DNA labelling intensity tended to be lower in chicken gonadal PGCs than in somatic cells, chromocenters remained visible at all stages, either as clearly defined foci near the nuclear center or embedded in the peripheral rim of heterochromatin (Fig. 4D). The nuclear distribution of the centromere protein CENP‑T did not differ between germ cells and somatic cells (data not shown); however, CENP‑T foci tended to be smaller and fainter in the former, suggesting chromatin decondensation, possibly resulting from the declustering of pericentromeres, akin to the occurrence in mouse PGCs [57]. We estimated chromatin density in gonadal PGC nuclei by measuring the fluorescence intensity of DNA counterstaining per unit area of nucleus section. For both sexes and at all stages, germ cell chromatin appeared about twofold less dense than somatic cell chromatin (Fig. 4E). Therefore, we analyzed the ultrastructure of gonadal cell nuclei using transmission electron microscopy in 14-day-old chick embryos (Fig. 4F). Germ cell nucleoplasm appeared less electron-dense compared with somatic cell nucleoplasm and lacked a discernible dense chromatin layer beneath the nuclear envelope. This pattern was similar to our previous observation in cPGCs [30], as well as observations of PGCs settling in the genital ridges of chicken embryos [59]. Thus, low-density fluorescent DNA staining in germ cell nuclei corresponded to low chromatin compactness.

### Establishing the germ-specific epigenetic signature during embryogenesis

**Fig. 5:**
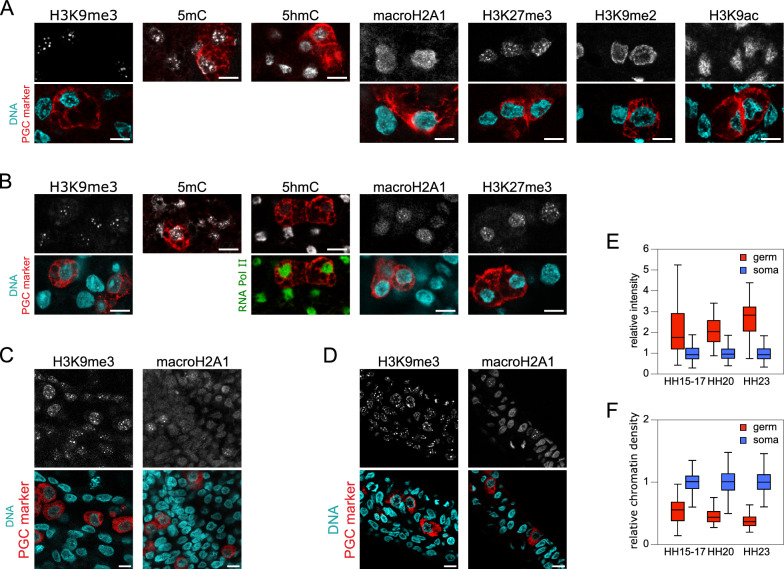
Setting of the germ-specific epigenetic signature during chicken embryo early development. Immunodetection of histone PTMs, macroH2A1, 5mC, and 5hmC (gray) in chicken embryos. Representative nuclei of PGCs identified with a germ cell marker (red) and somatic surrounding cells are shown. For 5hmC, additional labelling of RNA pol II was performed to locate nuclei when necessary. DNA staining (cyan). Scale bar: 10 µm. **A** In the blastoderm (stage EG&K X). **B** In the germinal crescent (stage HH4). **C** In blood vessels near the head (stage HH13). **D** At the genital ridges (stage HH15-17). **E** Quantification of fluorescence intensity for H3K9me3 in germ and somatic cell nuclei in stage HH15 to HH23 embryos. Number of analyzed nuclei in HH15-17, HH20, and HH23 embryos (two embryos per stage): 39, 46, and 54 germ cells and 157, 144, and 259 somatic cells, respectively. **F** Quantification of chromatin density (intensity of the DNA stain by unit area) in germ and somatic cell nuclei, for the same stage HH15 to HH23 embryos

We attempted to determine when the distinctive epigenetic profile observed in gonadal PGCs emerged during early embryo development. Initially, we examined PGCs at the blastoderm center in unincubated eggs (stage EG&K X–XII embryos). At this stage, PGC nuclei were indistinguishable from blastodermal somatic cell nuclei (Fig. 5A). H3K9me3, H3K27me3, and 5mC primarily localized at chromocenters, whereas H3K9me2, H3K9ac, 5hmC, and macroH2A1 exhibited a more homogeneous distribution. By the HH4 stage (Fig. 5B), when PGCs had relocated to the germinal crescent, their nuclei displayed a similar pattern to somatic cell nuclei for H3K9me3, H3K27me3, and 5mC, with macroH2A1 more frequently exhibiting foci in PGCs. Remarkably, about 61% of PGC nuclei (46 cells from 6 embryos) showed lower 5hmC richness compared with the surrounding somatic cell nuclei. By the HH13 stage (Fig. 5C), as PGCs began migration through the vascular system, some displayed features of the distinctive epigenetic profile, i.e., high H3K9me3 levels at diffuse regions outside chromocenters and less intense DNA staining of their nuclei compared with surrounding cell nuclei, suggesting chromatin decondensation. The macroH2A1 pattern with large foci was occasionally observed. Examining PGCs newly arrived at the genital ridges from the HH15 to HH23 stage, we found that the PGC-specific macroH2A1 nuclear pattern was present in most cells (Fig. 5D). Quantification of H3K9me3 labelling (as previously performed on the gonads) revealed significantly higher modification levels in PGCs compared to the surrounding somatic cells, with an increase over time during this period (Fig. 5E) and beyond until the maximum level was reached in the gonads. Concurrently, chromatin density in PGC nuclei decreased (Fig. 5F). Therefore, the first elements of the PGC-specific epigenetic signature appear early during embryonic development, preceding PGC migration in the bloodstream, with subsequent steps occurring progressively during migration and in the differentiating gonads.

### Genome-wide profiling of H3K9me3 in chicken PGCs

H3K9me3 global enrichment in chicken germ versus somatic cells is a striking distinctive feature of chicken PGCs compared to mammalian PGCs. It is also an uncommon epigenome feature, since observed H3K9me3 levels are generally similar in all cell types of a species, the modification being mostly present in constitutive heterochromatin such as PCH. However, fluorescence immunodetection did not suggest that the enrichment of H3K9me3 in chicken PGCs compared to somatic cells was located at PCH. To investigate precisely where this enrichment took place in the genome of chicken PGCs, we performed chromatin immunoprecipitation followed by high-throughput sequencing (ChIP-seq). We aimed at studying PGCs before they underwent sexual differentiation in the gonads. The low number of germ cells at migrating or early gonadal stages made the use of freshly isolated cells technically difficult. Consequently, as we had shown that cPGC maintained the epigenomic features of embryonic PGCs, we chose to analyze cPGCs versus ESCs, the in vitro derivatives of early embryonic germ and somatic cells. Studying such homogeneous cell populations also sharpened the identification of germ-specific features using combined epigenome and transcriptome analysis.

**Fig. 6:**
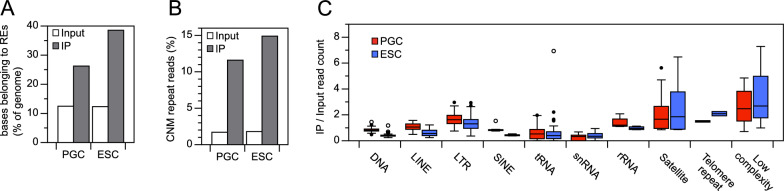
H3K9me3 at repeated elements in chicken PGCs compared with ESCs. **A** Overall enrichment for repeated elements (REs) in H3K9me3-immunoprecipitated and input chromatin. Counts of bases belonging to REs are expressed relative to the total base count in sequencing libraries. **B** Enrichment of CNM repeats in H3K9me3-immunoprecipitated and input chromatin. Counts of CNM repeat containing reads are expressed as a percentage of the total read count in sequencing libraries. **C** H3K9me3 enrichment for the major categories of REs identified using RepeatMasker. Read counts in IP relative to input chromatin libraries were calculated for each element type prior to grouping by class. *DNA* transposons, *LINE* long interspersed nuclear elements, *LTR* long terminal repeat containing elements, *SINE* short interspersed nuclear elements

Initially, we determined whether the modification was enriched at repetitive sequences, given that H3K9me3 is present in constitutive heterochromatin in chickens [30, 60]. Evaluating the proportion of repeated element sequences in the immunoprecipitated DNA, we found that approximately 26% of sequenced bases were located in repeated elements for cPGCs compared with around 39% for ESCs (Fig. 6A). This indicates that the higher global H3K9me3 level in cPGCs did not result from preferential increase of enrichment at repetitive sequences. To estimate H3K9me3 enrichment at PCH, we examined sequences of the chicken nuclear membrane (CNM) repeat, enriched at PCH in chickens [60]. The proportion of CNM-containing reads in the immunoprecipitated DNA was approximately 12% and 15% for cPGCs and ESCs, respectively (Fig. 6B), indicating that H3K9me3 was not preferentially concentrated at PCH in germ cells relative to somatic cells. This finding aligns with our observations regarding the nuclear localization of H3K9me3, suggesting that the enrichment in PGCs, in comparison to somatic cells, is not primarily concentrated at PCH but rather distributed across other genomic regions. Analyzing the presence of H3K9me3 in different subfamilies of RepeatMasker-annotated elements in the chicken genome (Fig. 6C), we found that low-complexity sequences, satellites, and telomeric sequences were highly enriched, consistent with their frequent localization in constitutive heterochromatin, whereas transfer RNAs, short interspersed nuclear elements, and small nuclear RNAs exhibited low enrichment. Interestingly, transposons (named “DNA” in RepeatMasker), long interspersed nuclear elements, long terminal repeat-containing elements, and short interspersed nuclear elements were slightly more enriched in cPGCs than in ESCs, implying enhanced repression of potentially mobile elements in germ cells.

**Fig. 7:**
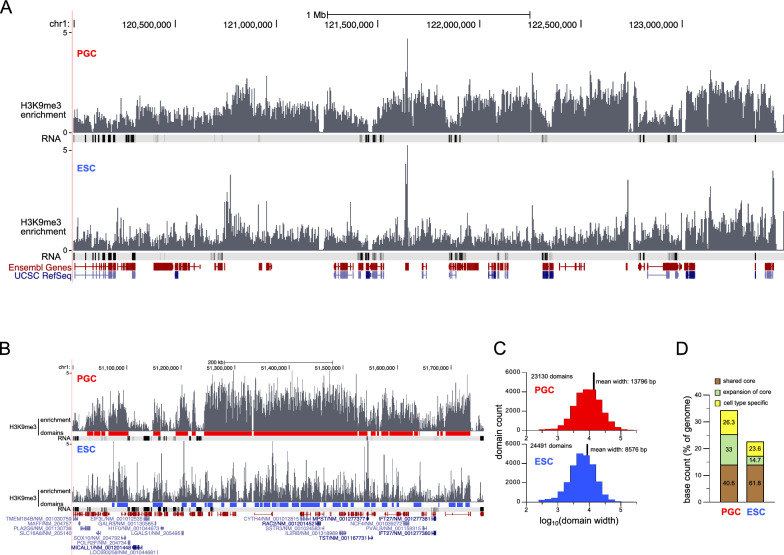
H3K9me3 domain characteristics in chicken PGCs compared with ESCs. **A** Genome browser view (UCSC) of H3K9me3 enrichment profile at a representative region of chicken chromosome 1. Gene positions (from Ensembl and UCSC RefSeq) and expression levels (RNA density track from RNA-seq analysis) are indicated below. **B** H3K9me3 domain mapping at a region exhibiting contrasted enrichment in PGCs and ESCs. **C** Counts and size distributions of domains. **D** Assessment of domain position similarity between PGCs and ESCs. For each cell type, the counts of bases covered by domains (expressed as genome percentages) are categorized into three classes, and the percentage of bases in each class is indicated on the bars. The difference in class distribution between the two cell types is highly significant (p < 2.2 × 10^−16^ based on the chi-squared test)

Next, we studied H3K9me3 distribution in the nonrepetitive parts of the genome using uniquely mapping sequencing reads (refer to “Methods” section for the rationale). Plotting the enrichment along the genome revealed the homogeneous presence of H3K9me3 at a medium level rather than localized sharp peaks above a low background (Fig. 7A). Notably, larger domains with distinct borders, indicating heightened enrichment over extended distances, were more prevalent in cPGCs. At numerous loci, the fold enrichment over background was higher in PGCs than in ESCs, despite no discernible difference in the local gene expression level. We implemented a custom peak-calling procedure, based on available tools, to delineate these H3K9me3 domains (Fig. 7B). In cPGCs, the domains were less numerous than in ESCs (23,130 vs. 24,491, respectively) but exhibited greater width (Fig. 7C). Ultimately, the proportion of the nonrepetitive genome covered by H3K9me3 domains was more extensive in cPGCs, with approximately 34% of the bases contained in domains, as opposed to around 22% in ESCs (Fig. 7D). To assess similarities in H3K9me3 domain locations between the two cell types, we examined the number of bases in cell type-specific or shared domains (Fig. 7D). Notably, most bases belonged to shared domains, with cPGCs displaying a high proportion of bases in expansions of the core of the shared domains. Many peaks present in ESCs appeared higher and broader in cPGCs, coalescing to form wide domains.

### Functional role of H3K9me3 domains

**Fig. 8:**
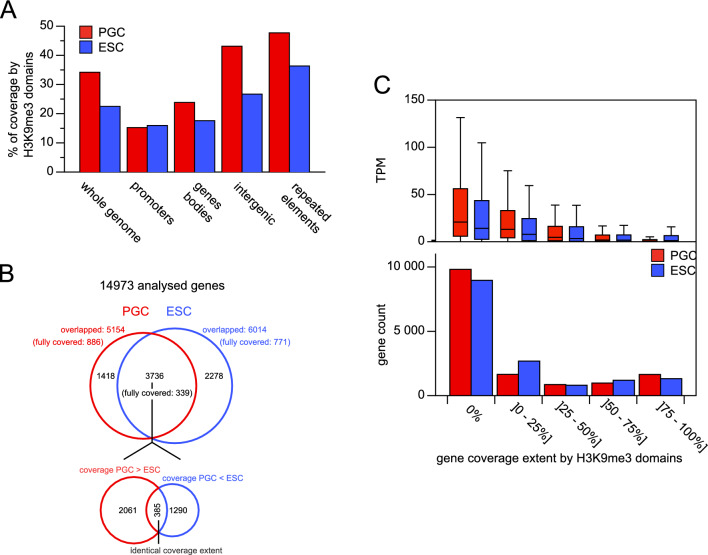
H3K9me3 domain positions relative to genes and transcriptional activity. **A** Coverage of genome functional regions by H3K9me3 domains. **B** Number of genes overlapped at least partially by H3K9me3 domains. **C** Expression levels of genes according to their coverage extent by H3K9me3 domains. For each coverage range class, the expression level (TPM) distribution of the corresponding genes is plotted above. TPM distributions of PGCs and ESCs are highly significantly different for all classes excepted the 50% to 75% class (p < 0.001 based on the Wilcoxon–Mann–Whitney test)

We categorized various functional regions based on chicken genome annotation and calculated their mean coverage by H3K9me3 domains. Consistent with the recognized role of H3K9me3 in heterochromatin, the highest coverage values were found in the most transcriptionally inactive regions, specifically intergenic regions and repeated elements (Fig. 8A). A comparison of results between cPGCs and ESCs revealed higher coverage in cPGCs for most types of regions (1.3 to 1.6-fold higher coverage), suggesting that the expansion of domains in cPGCs, relative to ESCs, was not limited to specific functional genome segments. Notably, promoters did not exhibit greater coverage based on H3K9me3 domains in cPGCs compared with ESCs. This indicates that the global augmentation of H3K9me3 in cPGCs did not correspond to an increased repressive presence at proximal regulatory sequences.

To determine whether the modification was involved in gene repression, we precisely examined how genes were overlapped by H3K9me3 domains and whether this correlated with their expression status. For each gene, we calculated the fraction of the locus covered by H3K9me3 domains and assessed the expression level using TPMs from the RNA-seq analyses on cPGCs and ESCs (Additional file 4: Table S3). Among the 14,973 genes considered in the analyses, the number of genes at least partially overlapped by a domain was lower in cPGCs than in ESCs (40% vs. 34%, respectively). However, genes affected by domains in both cell types exhibited higher coverage in cPGCs (Fig. 8B), aligning with the global extension of peaks in cPGCs compared with ESCs. For each cell type, we categorized genes into five groups based on the percentage of the locus covered by H3K9me3 domains and compared the TPM distributions in these groups (Fig. 8C). First, genes with greater coverage by domains exhibited lower expression levels, consistent with the involvement of H3K9me3 in transcriptional repression. Second, for high-coverage categories, genes with similar coverage tended to be more repressed in cPGCs compared with ESCs, indicating that genes with concentrated H3K9me3 were more efficiently repressed in cPGCs.

Finally, we conducted the functional analysis of genes extensively covered by H3K9me3 domains, selecting genes that were repressed (TPM lower than or equal to the first quartile of the TPM distribution) and covered by H3K9me3 domains at a minimum of 75% of their locus. In both cell types, this gene subset was significantly enriched in genes encoding components of the cell membrane, including transmembrane proteins such as G-protein coupled receptors (Additional file 5: gene lists A and B). Genes belonging to this functional family were occasionally localized in regions enriched with H3K9me3 domains, as observed for *SSTR3*, *RAC2*, and *IL2RB* (Fig. 7B). When selecting genes with a large H3K9me3 overlap (≥ 75%) in PGCs and a low H3K9me3 overlap (≤ 10%) in ESCs, we identified genes encoding the same protein types (Additional file 5: gene list C). Thus, most H3K9me3 is deposited in the same genome regions in cPGCs and ESCs, and the heightened coverage in cPGCs corresponds to the expansion of domains in these regions rather than the coverage of different, germ-specific regions.

## Discussion

In this study, we investigated the global DNA methylation and histone PTMs of chicken germ cells in vivo throughout early embryonic development. We showed that chicken PGCs begin to exhibit a distinct global epigenetic profile distinct from surrounding somatic cells before migrating into the bloodstream, progressively acquiring a specific and enduring epigenetic signature. Moreover, we found that prominent and conserved characteristics of the mammalian PGC signature were absent in chicken PGCs. Contrary to widespread genome-wide demethylation observed in mammals, 5mC levels remained high in embryonic gonadal germ cells. In previous studies, 5mC was detected in chicken gonadal PGCs by immunodetection [55], immunoprecipitation of methylated DNA [61], and genome-wide DNA methylation sequencing [62]. Our analysis of 5mC levels in PGCs relative to surrounding gonadal somatic cells confirms 5mC’s presence in PGCs, specifically at higher levels than those in somatic cells. Notably, the downregulation of genes involved in the loss of 5mC in mice was not observed in chicken PGCs. Furthermore, the low level of H3K9me2 and transient increase of H3K27me3 described for mammals were not observed in gonadal chicken PGCs. These global changes in histone PTMs during mammalian PGC epigenome reprogramming have been hypothesized to be connected to DNA demethylation. Specifically, it was proposed that H3K9me2, associated with the DNA methylation repressive pathway, would be replaced by H3K27me3, linked to the facultative heterochromatin/Polycomb repressive pathway [3]. Given the absence of genome-wide DNA demethylation in chicken PGCs, it is unsurprising that large-scale changes of these histone PTMs do not occur in this context.

Chicken PGCs exhibited certain chromatin characteristics observed in mammalian PGCs. Notably, the level of 5hmC was very low, akin to mammalian PGCs after epigenome reprogramming [9, 54, 63]. In mouse PGCs, after a transient enrichment of 5hmC resulting from 5mC conversion, 5hmC is lost from uniquely mapped genome regions, and transiently visible at chromocenters, being relocalized to repetitive elements. The scarcity of 5hmC in mouse PGCs may be attributed to a lack of 5mC availability for conversion. The observed reduction in *TET* gene expression levels or inhibition of 5mC conversion may be factors preventing the appearance of 5hmC in chicken PGCs despite high levels of 5mC.

Another shared feature with mammalian PGCs is a global chromatin structural organization distinct from that of somatic cells. Although chromocenters did not disappear, as initially suggested but not always confirmed in previous studies on mouse PGCs [4, 8, 10, 57], we observed macroH2A1’s relocation to chromocenters in chicken PGCs, eventually depleting from the nucleus, consistent with findings in mammals [4, 20]. Given that macroH2A is a global genome stabilizer (including repeated elements) and tends to prevent dedifferentiation during somatic cell reprogramming in mice (reviewed in [64]), its relocation to PCH indicates profound changes in the chromatin architecture of chicken PGCs, reflected by apparent low chromatin compactness, which is also observed in mouse PGCs [10].

Notably, chicken PGCs possess a germ-specific epigenetic characteristic absent in mammalian PGCs. They exhibit a high global level of H3K9me3 compared with somatic cell types. The enrichment begins when PGCs migrate into the bloodstream, intensifies upon arrival at genital ridges, and persists during gonadal proliferation. ChIP-seq profiling of H3K9me3 enrichment along the genome in cPGCs revealed that the modification is distributed as large domains located preferentially on transcriptionally inactive regions. Analysis of H3K9me3 distribution in ESCs, the most closely related somatic cell type, indicated the presence of similar domains located on the same types of genes. Previously, large H3K9me3 domains were found to be transiently deployed in murine germ-layer cells to repress genes associated with mature cell function [65], including numerous plasma membrane-related genes, similar to chicken PGCs. Our analysis suggests that the global enrichment of H3K9me3 in chicken PGCs results from increased coverage of inactive regions and not from a germ-restricted type of repression.

In mammals, the epigenetic signature of PGCs shares similarities with PSC chromatin, including low 5mC and 5hmC levels and diminished heterochromatin histone PTMs. This resemblance is likely because, following their specification from epiblast cells, migrating PGCs reacquire and retain some pluripotency characteristics [14]. In contrast, we have demonstrated that chicken migrating PGCs (both in vivo and cultured) and gonadal PGCs differ from chicken PSCs. Importantly, the germ-specific epigenetic profile is not initially present; blastoderm PGCs are indistinguishable from neighboring somatic PSCs in terms of the epigenetic marks that later contribute to their specific profile. Consequently, chicken PGCs shed the PSC-like signature they initially possess to acquire a germ-specific profile distinct from differentiated somatic cells. This change in the epigenetic signature, stable even when migrating PGCs are isolated and cultured, is likely related to germ cell function.

Several roles have been proposed for the epigenomic reprogramming in mouse PGCs [66]. The primary role involves removing parental imprints inherited by the embryo from gametes, by a genome-wide demethylation which would ensure the erasure of locus-specific imprints. As no parental imprinting is observed in chickens [67, 68], the need for DNA demethylation is obviated, explaining the absence of global 5mC loss and associated histone PTM changes. Another role in mammals may be to erase potential epigenetic barriers inherited from somatic epiblast cells, from which PGCs originate. However, chicken germ cells are segregated early in development from cells that did not acquire a somatic fate. Therefore, the erasure of restrictive marks may be dispensable, necessary only in species using the induction specification mode, such as mammalian species. Thus, the characteristics of chicken epigenetics and PGC specification may explain why chicken PGCs do not undergo the DNA methylation-related steps observed in mammalian epigenetic reprogramming.

As many mouse PGC chromatin characteristics are thought to be necessary for, or consequences of, genome demethylation, the existence of some of them (e.g., low chromatin density and 5hmC levels) in chicken PGCs, where they cannot be linked to DNA demethylation, raises questions about their function in germ cells. A study on mouse germ cells showed that chromatin decondensation (euchromatinization) persists beyond the canonical epigenetic reprogramming period, even when DNA methylation has been restored in gonadal stem cells [10]. This suggests that the decompacted state may play a role in itself, beyond merely facilitating DNA demethylation or as a direct consequence of 5mC’s absence. Interestingly, during the DNA demethylation process in mice, the open genome is protected from spurious gene transcription through various mechanisms, including elevated H3K27me3 levels. Subsequently, an expansion of H3K9me3 in broad domains is observed, albeit without major changes in modification abundance, in contrast to chicken PGCs [10, 69]. In chicken PGCs, the protection of the decondensed genome may be maintained continuously through the reinforcement of the H3K9me3-dependent repressive pathway. Indeed, we observed an increase in H3K9me3 levels parallel to the decrease in chromatin density. Additionally, the enrichment profile for H3K9me3 shows that the modification is globally augmented on inactive genomic regions embedded in repressive chromatin. The enrichment may also protect against the excessive activity of mobile elements, which exhibit greater modification in chicken PGCs compared with somatic cells according to the comparison with ESCs. Such a role for H3K9me3 have been suggested in the mouse and human germ line to compensate for the loss of regular repression through DNA methylation [69, 70].

In chicken PGCs, the global H3K9me3 level begins to increase after the relocation of macroH2A1 at PCH. This relocation, coupled with global loss, results in a reduced presence in other genomic compartments. We propose that macroH2A depletion triggers the reorganization of chromatin into a more open configuration in both chicken and mammalian PGCs. Indeed, macroH2A knockout in mouse cells leads to diffuse distribution of H3K9me3 and loss of heterochromatin observed through MET [64, 71], resembling the pattern observed in chicken PGCs. As macroH2A1 controls interactions between chromatin and the nuclear lamina, its depletion and relocation to PCH in PGCs could be responsible for the erasure of lamin-associated domains observed during the euchromatinization process [10], and thus for the heterochromatin layer’s disappearance at the nuclear periphery.

## Conclusions

In summary, the epigenetic signature of chicken PGCs is gradually established during migration and remains stable in the gonads. Unlike in mammals, there is no loss of the marks associated with the DNA methylation/H3K9 methylation/HP1 repressive pathway; instead, these elements are globally intensified, particularly in inactive or repressed genome regions. Despite the presence of these heterochromatin marks, the chromatin in chicken PGC nuclei appears decondensed, akin to mammalian nuclei. Additionally, relocation or depletion of macroH2A and 5hmC occurs, reflecting observations in mammalian PGCs. Given the absence of parental imprinting and DNA demethylation, the large-scale epigenetic events observed in chicken PGCs appear more as chromatin reconfiguration rather than *bona fide* reprogramming. Notably, the acquisition of a decondensed genome for germ cell development is a shared feature between mammals and birds, despite their differences in germ cell DNA methylation regulation and modes of specification. This underscores the functional importance of chromatin decondensation, the role of which in meiosis preparation warrants further investigation.

### Supplementary Information

**Additional file 1: Table S1.** Transcriptome analysis of chicken PGCs and ESC. Differential gene expression analysis of PGCs vs. ESCs was performed using DESeq2. TPM values were obtained using Salmon and processed as described in “Methods” section.

**Additional file 2**: **Table S2.** Expression of chicken pluripotency-associated, germinal, *HOX* and epigenetic modifier genes highlighted in the study. Log_2_ fold-changes (differential gene expression analysis of PGCs versus ESCs) and TPMs (transcript per million) are extracted form the transcriptome analysis results (Table S1).

**Additional file 3: Figure S1.** Histone K4 methylation and histone acetylation in the gonads of chicken embryos. **A** Immunodetection of H3K4me3 (gray) and germ cell marker (red) in tissue sections. DNA staining (cyan). Scale bar: 10 µm. Quantification of fluorescence intensity in germ and somatic cell nuclei is shown below. Number of analyzed nuclei in 6‑, 8‑, 10‑, and 14‑day-old embryos: for females, 39, 38, 26, and 52 germ cells and 101, 75, 75, and 70 somatic cells, respectively; for males, 32, 35, 33, and 55 germ cells and 125, 200, 75, and 149 somatic cells, respectively. **B** Immunodetection of H3K4me1 (gray) and germ cell marker (red) in tissue sections. DNA staining (cyan). Scale bar: 10 µm. Quantification of fluorescence intensity in germ and somatic cell nuclei is shown below. Number of analyzed nuclei in 6‑, 8‑, 10‑, and 14‑day-old embryos: for females, 43, 33, 38, and 42 germ cells and 151, 151, 75, and 53 somatic cells, respectively; for males, 38, 37, 35, and 29 germ cells and 166, 127, 102, and 76 somatic cells, respectively. **C** Immunodetection of H3K27ac (gray) and germ cell marker (red) in tissue sections. DNA staining (cyan). Scale bar: 10 µm. Quantification of fluorescence intensity in germ and somatic cell nuclei is shown below. Number of analyzed nuclei in 6‑, 8‑, 10‑, and 14‑day-old embryos: for females, 39, 31, 26, and 72 germ cells and 190, 372, 203, and 111 somatic cells, respectively; for males, 34, 37, 39, and 35 germ cells and 192, 152, 100, and 100 somatic cells, respectively. **D** Immunodetection of H3K9ac (gray) and germ cell marker (red) in tissue sections. DNA staining (cyan). Scale bar: 10 µm. Quantification of the fluorescence intensity in germ and somatic cell nuclei is shown below. Number of analyzed nuclei in 6‑, 8‑, 10‑, and 14‑day-old embryos: for females, 40, 52, 54, and 42 germ cells and 125, 225, 125, and 50 somatic cells, respectively; for males, 43, 32, 38, and 41 germ cells and 125, 125, 75, and 99 somatic cells, respectively.

**Additional file 4: Table S3.** Gene coverage by H3K9me3 domains. The extent of coverage was calculated for each gene locus including the promoter (i.e., − 500 bp from TSS to TTS) and expressed as a percentage of locus length.

**Additional file 5.** Functional annotation of genes preferentially overlapped by H3K9me3 domains. Analysis results from DAVID on subsets of genes are shown as clustering reports to group similar annotations. **Gene list A**. Genes with high overlap (≥ 75%) and weak expression (TPM < first quartile of TPM distribution) in PGCs. **Gene list B**. Genes with high overlap (≥ 75%) and weak expression (TPM < first quartile of TPM distribution) in ESCs. **Gene list C**. Genes with high overlap (≥ 75%) in PGC and low overlap (< 10%) in ESCs.

## Data Availability

The datasets supporting the conclusions of this article are included within the article and its additional files. The datasets supporting the conclusions of this article are available from the NCBI Gene Expression Omnibus (http://www.ncbi.nlm.nih.gov/geo/) through GEO Series accession number GSE248768.

## Abbreviations

5mC: 5‑methylcytosine
5hmC: 5‑hydroxymethylcytosine
BC: Blastodermal cell
CEF: Chicken embryonic fibroblast
ESC: Embryonic stem cell
H3K9me3: Histone H3 lysine 9 trimethylation
H3K9me2: Histone H3 lysine 9 dimethylation
H3K27me3: Histone H3 lysine 27 trimethylation
PCH: Pericentric heterochromatin
PGC: Primordial germ cell
