# Supplementary figures and images for: Reinforcement of repressive marks in the chicken primordial germ cell epigenetic signature: divergence from basal state resetting in mammals
Source: Epigenetics Chromatin. 2024 Apr 26;17:11. doi: 10.1186/s13072-024-00537-7 (PMC11046797; doi:10.1186/s13072-024-00537-7)

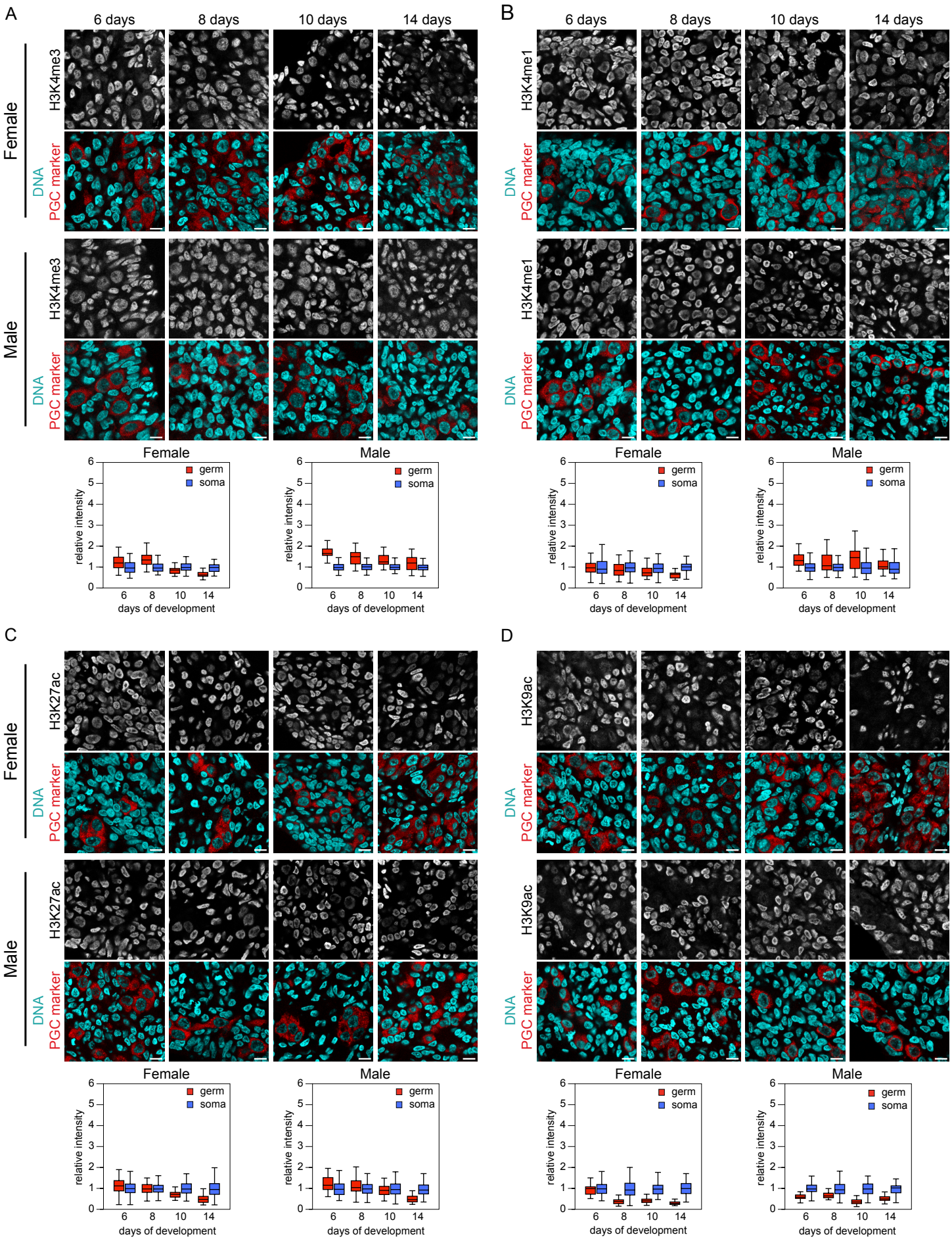

Supplement: Supplementary file 3 — Additional file 3: Figure S1. Histone K4 methylation and histone acetylation in the gonads of chicken embryos. A Immunodetection of H3K4me3 (gray) and germ cell marker (red) in tissue sections. DNA staining (cyan). Scale bar: 10 µm. Quantification of fluorescence intensity in germ and somatic cell nuclei is shown below. Number of analyzed nuclei in 6‑, 8‑, 10‑, and 14‑day-old embryos: for females, 39, 38, 26, and 52 germ cells and 101, 75, 75, and 70 somatic cells, respectively; for males, 32, 35, 33, and 55 germ cells and 125, 200, 75, and 149 somatic cells, respectively. B Immunodetection of H3K4me1 (gray) and germ cell marker (red) in tissue sections. DNA staining (cyan). Scale bar: 10 µm. Quantification of fluorescence intensity in germ and somatic cell nuclei is shown below. Number of analyzed nuclei in 6‑, 8‑, 10‑, and 14‑day-old embryos: for females, 43, 33, 38, and 42 germ cells and 151, 151, 75, and 53 somatic cells, respectively; for males, 38, 37, 35, and 29 germ cells and 166, 127, 102, and 76 somatic cells, respectively. C Immunodetection of H3K27ac (gray) and germ cell marker (red) in tissue sections. DNA staining (cyan). Scale bar: 10 µm. Quantification of fluorescence intensity in germ and somatic cell nuclei is shown below. Number of analyzed nuclei in 6‑, 8‑, 10‑, and 14‑day-old embryos: for females, 39, 31, 26, and 72 germ cells and 190, 372, 203, and 111 somatic cells, respectively; for males, 34, 37, 39, and 35 germ cells and 192, 152, 100, and 100 somatic cells, respectively. D Immunodetection of H3K9ac (gray) and germ cell marker (red) in tissue sections. DNA staining (cyan). Scale bar: 10 µm. Quantification of the fluorescence intensity in germ and somatic cell nuclei is shown below. Number of analyzed nuclei in 6‑, 8‑, 10‑, and 14‑day-old embryos: for females, 40, 52, 54, and 42 germ cells and 125, 225, 125, and 50 somatic cells, respectively; for males, 43, 32, 38, and 41 germ cells and 125, 125, 75, and 99 somatic cells, respectively. [file 13072_2024_537_MOESM3_ESM.pdf]
